# Metastatic Small-Cell Lung Cancer Presenting as Primary Adrenal Insufficiency

**DOI:** 10.1155/2020/7018619

**Published:** 2020-03-11

**Authors:** Shawn Esperti, Austen Stoelting, Nicolina Scibelli, David Moccia, Dveet Patel, Michael Haughton, Andrew Mangano

**Affiliations:** Department of Internal Medicine, Grand Strand Medical Center, Myrtle Beach, SC, USA

## Abstract

A 40-year-old male smoker with HIV was admitted for cough, hypotension, and abdominal pain for 5 days. Chest radiography showed a right lower lobe consolidation. CT of the chest, abdomen, and pelvis revealed paratracheal adenopathy, a 5.8 × 4.5 cm mass invading the right bronchus intermedius, and dense bilateral adrenal masses, measuring 5.4 × 4.0 cm on the right and 4.8 × 2.0 cm on the left. Laboratory studies showed white blood cell count of 18.5 K/mm^3^, sodium of 131 mmol/L, creatinine of 1.6 mg/dL, and CD4 count of 567 cells/mm^3^. The random morning cortisol level was 7.0 *μ*g/dL, the ACTH stimulation test yielded inappropriate response, and a random serum ACTH was elevated at 83.4 pg/mL. MRI brain revealed no pituitary adenoma confirming primary adrenal insufficiency. The adrenal CT washout study was consistent with solid mass content, concerning for metastasis. Bronchoscopy with endobronchial mass and paratracheal lymph node biopsy confirmed small-cell lung cancer (SCLC). Intravenous steroids, 100 mg hydrocortisone every 8 hours, improved his hypotension and abdominal pain. PET scan revealed metabolically active right paratracheal mass, right hilar mass, and bilateral adrenal masses. Treatment included palliative chemotherapy consisting of carboplatin/etoposide/atezolizumab and chest radiation. We present this novel case to demonstrate SCLC's ability to cause primary adrenal insufficiency, as well as evaluate clinical response to chemotherapeutics.

## 1. Introduction

Small-cell lung cancer (SCLC) is a neuroendocrine tumor highly associated with heavy tobacco use and represents about 15% of all primary lung cancers [1]. It is recognized as the most rapidly progressive lung cancer, with up to 60% of cases with metastatic disease at the time of diagnosis [2]. SCLC is distinguished from non-small-cell lung cancers due to its rapid mitotic rate with median survival of approximately 2–4 months when untreated and a 5-year survival rate in the range of 4%–5% when treated [3]. The most common sites of distant metastases are the liver, bone, brain, lung, and adrenal gland, respectively [3]. Although adrenal metastases comprise 6% of all SCLC metastases, there has been only one case report of primary adrenal insufficiency resulting from SCLC metastasis published to date [4]. We present this case to demonstrate SCLC's aggressive nature and ability to cause primary adrenal insufficiency with extensive bilateral adrenal metastases. We will also examine this patient's clinical response to chemotherapeutic agents.

## 2. Case Report

**Figure 1:**
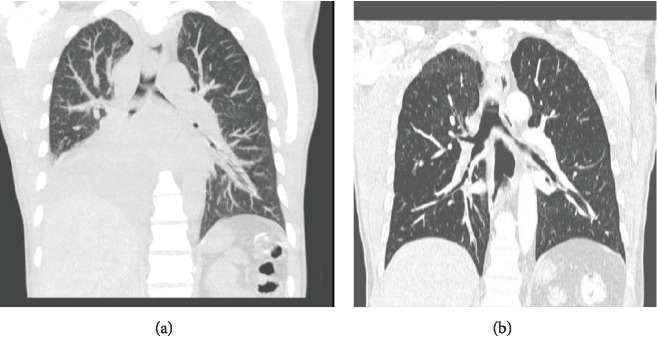
(a) Chest CT without contrast prior to chest radiation showing a 5.8 × 4.5 cm right infrahilar mass obstructing the bronchus intermedius with right paratracheal adenopathy. Postobstructive consolidation of the right lower lobe and patchy infiltration of the right middle lobe. (b) Chest CT with IV contrast postchest radiation revealing significant improvement in the right hilar lesion. There is slight thickening around the right main bronchus; the large soft tissue lesion has almost totally resolved with resolution of postobstructive changes.

**Figure 2:**
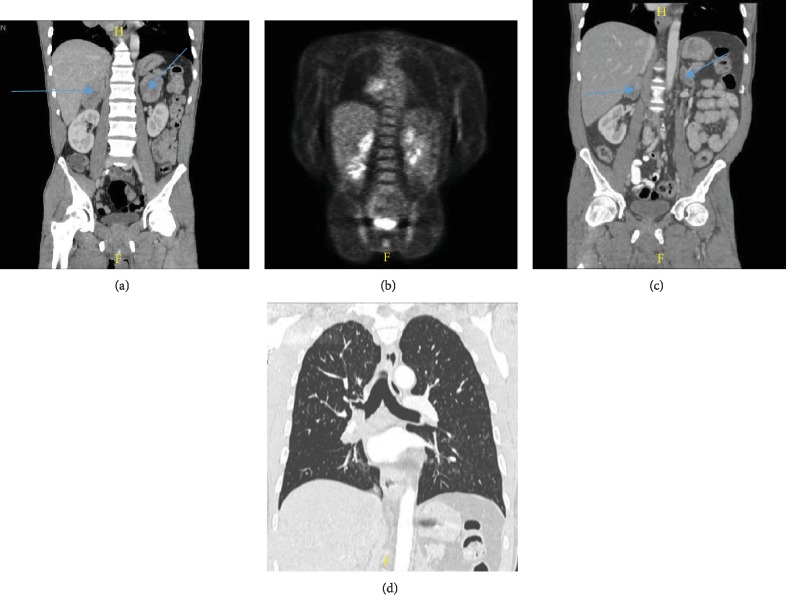
(a) CT abdomen and pelvis with IV contrast showing bilateral adrenal masses with the right measuring 5.4 × 4.0 cm and the left measuring 4.8 × 2.0 cm, highly concerning for metastatic disease. Arrows pointing to adrenal masses. (b) PET CT prior to chemotherapy showing metabolically active right hilar mass, metabolically active right paratracheal lymphadenopathy, decreased prominence of mediastinal adenopathy, and metabolically active bilateral adrenal masses consistent with metastatic disease. (c) CT abdomen and pelvis with PO and IV contrast demonstrates that both adrenal glands have significantly decreased in size, measuring 2.9 × 1.6 cm on the left and 3.4 × 2.0 cm on the right. Arrows pointing to adrenal masses. (d) CT of the chest revealing near-total resolution of the right hilar mass without postobstruction.

**Figure 3:**
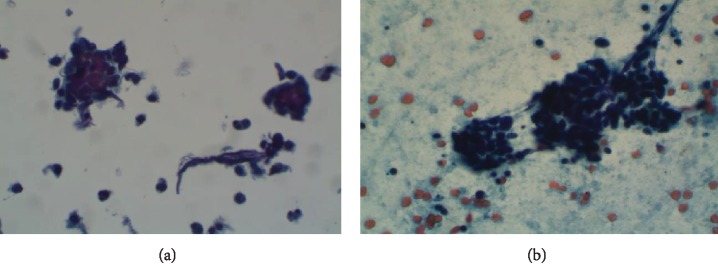
(a) Direct smears and thin prep revealing carcinoma in a neuroendocrine pattern of poorly cohesive cells with increased nuclear to cytoplasmic ratio with salt-and-pepper nuclei and nuclear molding of endobronchial mass tissue. (b) Direct smears and thin prep at high magnification revealing the same results of 4R lymph node biopsy.

A 40-year-old male with a past medical history of tobacco abuse and HIV presented to our hospital for cough, nausea, vomiting, and abdominal pain of 5-day duration. Upon arrival, the patient was afebrile, with a pulse rate of 104 and a blood pressure of 80/60. Chest radiography revealed a right lower lobe consolidation. Chest CT revealed right paratracheal adenopathy and a 5.8 × 4.5 cm mass occluding the bronchus intermedius (Figure 1(a)). CT of the abdomen and pelvis without contrast revealed dense bilateral adrenal masses, measuring 5.4 × 4.0 cm on the right and 4.8 × 2.0 cm on the left (Figure 2(a)). Laboratory studies were significant for a white blood cell count of 18.5 K/mm^3^, sodium of 131 mmol/L, creatinine of 1.6 mg/dL, and CD4 count of 567 cells/mm^3^. Due to concerns for sepsis, the patient was admitted to the hospitalist service, given IV fluids, and started on vancomycin and piperacillin/tazobactam empirically. Infectious workup included serum CMV, Influenza PCR, Mycoplasma and Bartonella IgG/IgM, Cryptococcus, Histoplasma, and Legionella urinary antigen that all returned negative. With ongoing hypotension while on antibiotics, a random morning cortisol level was ordered to evaluate for adrenal insufficiency. This returned at the lower limit of normal at 7.0 *μ*g/dL. The ACTH stimulation test displayed an inappropriate response with cortisol levels at 30 minutes and 60 minutes of 10.1 *μ*g/dL and 10.3 *μ*g/dL, respectively. Serum ACTH was elevated at 83.4 pg/mL. MRI brain revealed no pituitary adenoma confirming the diagnosis of primary adrenal insufficiency. Stress dose steroids consisting of 100 mg intravenous hydrocortisone every 8 hours resulted in resolution of hypotension and abdominal pain. Adrenal function stabilized on 15 mg of oral prednisone in the morning and 5 mg in the evening. The adrenal CT washout study showed precontrast densities measuring 27, 54, and 61 Hounsfield units on the precontrast, portal venous phase, and delayed imaging on the right, respectively. The left adrenal gland measured 32, 48, and 51 Hounsfield units on precontrast, postcontrast, and delayed imaging, respectively, both consistent with metastatic disease. Pathology from the endobronchial mass and 4R lymph node biopsy revealed poorly differentiated neuroendocrine carcinoma confirming SCLC (see Figure 3 below). The patient started inpatient radiation therapy to his lung mass followed by outpatient chemotherapy. Two rounds of palliative chest radiation with 8 Gy resulted in a significant decrease in the size of the primary mass and relief of obstruction (Figure 1(b)).

Outpatient PET scan revealed metabolically active right paratracheal mass, right hilar mass, and bilateral adrenal masses (Figure 2(b)). Chemotherapy consisted of carboplatin/etoposide/atezolizumab every three weeks for four cycles. The patient declined whole brain radiation due to risk of cognitive dysfunction. After two cycles of chemotherapy, follow-up CT scans revealed a 50% reduction in adrenal masses (Figure 2(c)) and near-total resolution of the right hilar mass without any postobstruction (Figure 2(d)).

**Figure 4:**
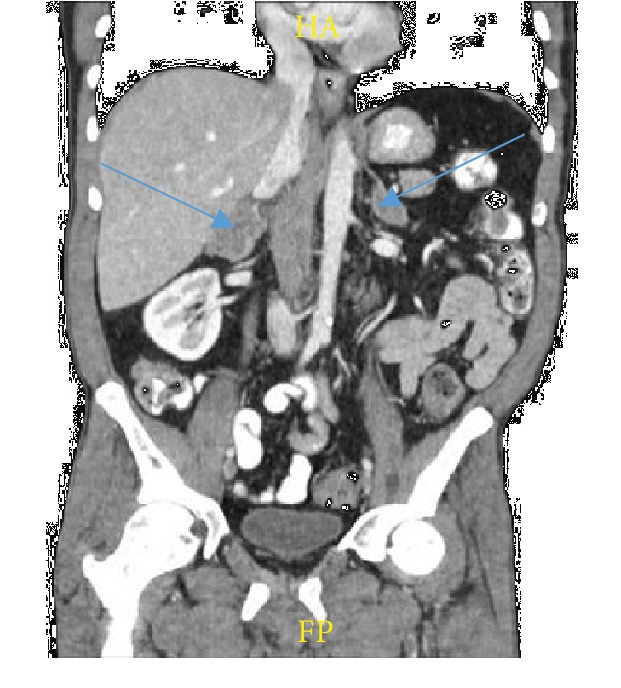
CT abdomen and pelvis with IV and PO contrast showing the right adrenal mass increased from 3.4 × 2.0 cm to 4.3 × 2.9 cm with more irregular margins. The left adrenal mass increased from 2.9 × 1.6 cm to 3.9 × 2.0 cm. Arrows pointing to adrenal masses.

The patient finished his final two cycles of chemotherapy and transitioned to maintenance atezolizumab. At two months of maintenance therapy follow-up staging, CT scan revealed a mixed response to therapy. The right adrenal mass increased from 3.4 × 2.0 cm to 4.3 × 2.9 cm with more irregular margins. The left adrenal mass increased from 2.9 × 1.6 cm to 3.9 × 2.0 cm (Figure 4) while the right lung soft tissue mass continued to decrease from 1.0 × 0.6 cm to 0.8 × 0.8 cm. After treatment failure, the patient was transitioned to hospice care.

## 3. Discussion

The adrenal glands have a rich sinusoidal blood supply providing a conduit for infection and metastasis [5]. Bilateral adrenal metastases at the time of diagnosis are found in 3% of SCLC patients [6], but many other etiologies must be considered. The differential diagnosis for bilateral adrenal masses remains broad and includes a spectrum of disorders including infectious causes, neoplastic causes, endocrinopathies, or even traumatic causes [7]. Infectious etiologies include mycobacterial and fungal infections. Disseminated mycobacterial tuberculosis, histoplasmosis, and blastomycosis have been reported frequently, and parasitic infections remain rare [8]. Metastasis is the most common malignancy, but also lymphoma, bilateral pheochromocytoma, adrenocortical carcinomas, and myelolipomas have presented as bilateral adrenal masses [9]. Long-standing uncontrolled congenital adrenal hyperplasia and macronodular hyperplasia are endocrine disorders that can present as bilateral adrenal masses [10]. Bilateral adrenal hemorrhage should be considered especially in the setting of trauma, sepsis with vasopressor usage, antiphospholipid syndrome, and the use of anticoagulants [11].

Once primary adrenal insufficiency was diagnosed with a low morning cortisol, elevated ACTH, inappropriate ACTH stimulation test, and MRI showing no central lesion, the adrenal masses were the likely etiology. Without any upper lobe densities and negative infectious workup, tuberculosis and fungal infection were unlikely. CT of the abdomen and pelvis revealed bilateral masses without evidence of necrosis or abscess that would suggest adrenal hemorrhage or infection. With a normal serum renin : aldosterone ratio and plasma metanephrines, functional adenoma and pheochromocytoma were ruled out. The adrenal CT washout study did not demonstrate any significant density changes consistent with solid metastatic disease. Once endobronchial biopsy of the right hilar mass and paratracheal nodes showed SCLC, coupled with increased adrenal gland metabolic activity on PET scan, it was reasonable to presume the adrenal masses were SCLC.

Although lung cancer does metastasize to the adrenal gland, adrenal insufficiency is rare, as greater than 90% of the functional cortex must be destroyed for this to occur [12]. In one retrospective study spanning 30 years with 464 patients with adrenal metastatic disease from various tumors, only five of these patients developed adrenal insufficiency [13]. Primary adrenal insufficiency in the setting of SCLC has been reported only in one case report in Japan [4]. This case highlights the aggressive potential of SCLC in a novel fashion. SCLC was able to obliterate the adrenal glands, by exploiting the body's vascular network for dissemination and nourishment for rapid growth.

## 4. Conclusion

Although exceedingly rare, metastatic SCLC should be considered in the differential diagnoses for primary adrenal insufficiency in patients with risk factors such as tobacco abuse and HIV. This case demonstrates the aggressive nature of SCLC, and its potential to cause significant adrenal tissue destruction leading to primary adrenal insufficiency.
